# Care Transitions in Older Colorectal Surgery Patients: System, Provider, and Patient Insights

**DOI:** 10.1093/geroni/igaf122.3553

**Published:** 2025-12-31

**Authors:** Mark Iskandar, Ferhat Yildiz, Christine Ritchie, Sevdenur Cizginer

**Affiliations:** Central Michigan University, Mount Pleasant, Michigan, United States; Harvard Medical School, Boston, Massachusetts, United States; Massachusetts General Hospital, Boston, Massachusetts, United States; Massachusetts General Hospital, Boston, Massachusetts, United States

## Abstract

Older adults (age ≥65) undergoing colorectal surgery are at risk of medication errors, complications, and comorbidity exacerbation during care transitions. In this population, approximately 31% experience postoperative complications, with a 1.8-fold increase in functional decline and a 15% chance of readmission within 30 days (May, 2025). Given colorectal surgeries in older adults are projected to increase 50% by 2040, improving post-surgical transitions is important (Xie, 2025). From September 2023 to April 2024, we conducted semi-structured interviews with 42 stakeholders, including older surgical patients, caregivers, and providers from 12 disciplines. Using an inductive–deductive approach, we developed a preliminary codebook organized around system, provider, and patient domains containing codes for challenges, determinants, and solutions. Two coders independently applied and refined the codebook during thematic analysis, resolving discrepancies through discussion. Thematic analysis of transcripts revealed gaps and potential solutions in care transitions. At provider level, challenges included disorganized handoffs, poor medical team continuity, and limited collaboration with primary care. At system level, barriers included inadequate interdisciplinary coordination, insufficient post discharge resources, and difficulty accessing specialty services. At patient level, challenges included medication adherence, understanding discharge instructions, and avoiding readmission. Proposed solutions included stronger provider communication, enhanced education and support for patients and caregivers, and early discharge planning initiated before surgery, extending to virtual follow-ups post-discharge and connection to community resources. Improving transitions requires a multilevel approach that addresses communication gaps, system barriers, and patient vulnerabilities. These stakeholder-identified solutions highlight feasible interventions that could improve care quality and outcomes in this growing population.

